# Distinct Age-Related Clinical Features and Risk Assessment in Chinese With Chronic Lymphocytic Leukemia

**DOI:** 10.3389/fonc.2022.885150

**Published:** 2022-05-12

**Authors:** Zheng Tian, Ming Liu, Xiaosheng Fang, Xiangxiang Zhou, Peipei Li, Ying Li, Lingyan Zhang, Fang Liu, Ya Zhang, Xin Wang

**Affiliations:** ^1^ Department of Hematology, Shandong Provincial Hospital, Cheeloo College of Medicine, Shandong University, Jinan, China; ^2^ Department of Hematology, Shandong Provincial Hospital Affiliated to Shandong First Medical University, Jinan, China; ^3^ School of Medicine, Shandong University, Jinan, China; ^4^ Shandong Provincial Engineering Research Center of Lymphoma, Jinan, China; ^5^ Branch of National Clinical Research Center for Hematologic Diseases, Jinan, China; ^6^ National Clinical Research Center for Hematologic Diseases, the First Affiliated Hospital of Soochow University, Suzhou, China; ^7^ Campbell Family Mental Health Research Institute, Centre for Addiction and Mental Health, Department of Psychiatry, University of Toronto, Toronto, ON, Canada

**Keywords:** chronic lymphocytic leukemia, age, prognosis, real world observation, risk score

## Abstract

The biological and clinical features of chronic lymphocytic leukemia (CLL) exhibited profound heterogeneity across Chinese and patients of predominately European descent. However, the age-related peculiarities and risk assessment of Chinese CLL patients remained ill-defined. The present study demonstrated that CLL patients were characterized by the earlier age at onset in China (median age at diagnosis: 63 years old) than in the United States (median age at diagnosis: 69 years old). Young patients from Shandong Provincial Hospital CLL database displayed prolonged overall survival than the Surveillance, Epidemiology, and End Results cohort. Furthermore, among Chinese CLL patients, young patients showed an increased relapse rate compared with elderly patients. To optimize the risk assessment of CLL patients, novel risk score models named *PR-Score* and *HBG-Score* were developed for predicting the outcomes of young and elderly CLL patients respectively. The neonatal survival prediction systems were superior to international prognostic index for CLL (CLL-IPI) and Binet stage in assessing the overall survival and progression free survival of CLL patients. The analyses highlighted refinement of risk evaluation for CLL patients in different age groups, providing insights into individualized diagnosis and treatment of CLL.

## Introduction

Chronic lymphocytic leukemia (CLL), the most prevalent adult leukemia in Western countries, is characterized by proliferation and accumulation of mature B lymphocytes in peripheral blood, bone marrow, spleen and lymph nodes (1). The diagnosis and treatment of patients with CLL are transforming rapidly as the knowledge of CLL advances. National Comprehensive Cancer Network guidelines indicate that the diagnosis of CLL is based on peripheral blood cell count, blood smear, and flow cytometric immunophenotyping (1). Although Rai and Binet staging systems are commonly used for making decisions on treatment initiation, they are not satisfactory as long-term prognostic indicators (2). A number of molecular prognostic markers including somatic mutation status of immunoglobulin heavy chain variable region genes (IGHV), expression of zeta-associated protein-70, and expression of CD38 contribute to the prediction of prognosis in CLL (3–5). Before the introduction of novel molecular-targeted agents, CLL patients usually received chemoimmunotherapy regimens. Young patients with CLL usually received high-intensity chemoimmunotherapy with fludarabine, cyclophosphamide, and rituximab regimen or bendamustine and rituximab regimen (6, 7). However, elderly patients were treated with low-intensity chemoimmunotherapy because of the poor tolerance to chemoimmunotherapy (8–10). Novel molecular-targeted agents including ibrutinib, duvelisib, venetoclax, acalabrutinib and idelalisib have provided more options to CLL patients (11–13).

The incidence of CLL constantly increases with age, and the median age at onset of CLL was between 67 and 72 years old in the United States (US) (14, 15). Because of improved quality of life and advanced medical technology, the proportion of young CLL patients with early symptoms is increasing. However, young CLL patients are different from elderly CLL patients in multiple aspects. For example, single-nucleotide polymorphisms at 34 reported loci associated with increased genetic risk are more frequent in young CLL patients (16). And familial CLL patients developed the disease at a younger age compared with sporadic patients (17). Although ageing is an important risk factor for various cancers, the mechanism which links ageing and CLL remains unclear (18).

The epidemiological characteristics of CLL presented profound heterogeneity across East Asia and Western countries. The age adjusted incidence rate of CLL in East Asians was between 0.3 and 0.4 per 100,000, which was up to 10 times lower than that in persons of predominately European descent. Moreover, Asians with CLL are featured by the earlier age at onset compared with persons of predominately European descent. The median age at diagnosis of CLL ranged from 58 to 62 years old in China, and the median age at diagnosis of Chinese CLL patients included in this study was 63 years old (14). Moreover, the present study revealed the different survival profiles of young CLL patients between China and Western countries. Nevertheless, the relationship between age at onset and other clinical characteristics of Chinese CLL patients remained ill-defined. This investigation analyzed the divergent clinical characteristics and prognostic factors among young (<60 years old) and elderly (≥60 years old) Chinese CLL patients. The neonatal prognostic model named *PR-Score* was developed for predicting the outcomes of young CLL patients, and the *HBG-Score* was developed for predicting the outcomes of elderly CLL patients. This knowledge is expected to refine the specific risk stratification systems for CLL patients and optimize treatment regimens for CLL patients in different age groups.

## Materials and Methods

### Patients

A total of 601 CLL patients who were treated in Shandong Provincial Hospital between October 2010 and October 2021 were assessed from the Shandong Provincial Hospital CLL (SPHCLL) database. The diagnosis of CLL was based on the International Workshop on CLL-National Cancer Institute criteria. The cut-off of young and elderly patients was defined as 60 years old, a cut-off that separated patients with the most significant differences. CLL patients <60 years old were divided into the young group and CLL patients ≥60 years old were divided into the elderly group. The survival data of 2580 CLL patients registered in the Surveillance, Epidemiology, and End Results (SEER) database between 2010 and 2018 were obtained with SEER*Stat software version 8.1.5. The grouping criteria mentioned above for the Chinese cohort were also applied to the SEER cohort.

### Data Collection

Clinical-pathological parameters including hemoglobin (Hb), albumin, lactic dehydrogenase (LDH) and β2-microglobulin were accessed from the hospital-based laboratory service within 24 hours after the first admission. Fluorescence *in situ* hybridization (FISH) analysis was performed to detect del (17p13), del (11q22.3), del (13q) and trisomy 12. Determination of the IGHV mutational status was performed according to the guidelines for diagnosis and treatment of chronic lymphocytic leukemia/small lymphocytic lymphoma in China (2018 edition). Overall survival (OS) was defined as the time from the first diagnosis to the last follow-up or the time of death. Progression-free survival (PFS) was defined as the time from the first diagnosis to relapse, progression or death.

### Statistical Analyses

Data were analyzed by the IBM SPSS software version 23 and R software version 4.0.5. Comparisons of numerical variables between young and elderly CLL patients were performed by t tests for unpaired samples. Analyses of categorical variables were performed by χ² tests. Survival curves were constructed using the Kaplan-Meier method with the log-rank test. The univariate Cox regression analysis and multivariate Cox regression analysis were applied to evaluate the prognostic values of clinical-pathological features. For the multivariate analysis, we included variables whose *p*-values were less than 0.05 in the univariate analysis. The optimal cut-off points for risks scores were calculated using the “surv_cutpoint” function of the “survminer” R package. A value of *p* < 0.05 was defined as the borderline of statistical significance.

## Results

### Age Composition of SPHCLL and SEER Patients

**Figure 1:**
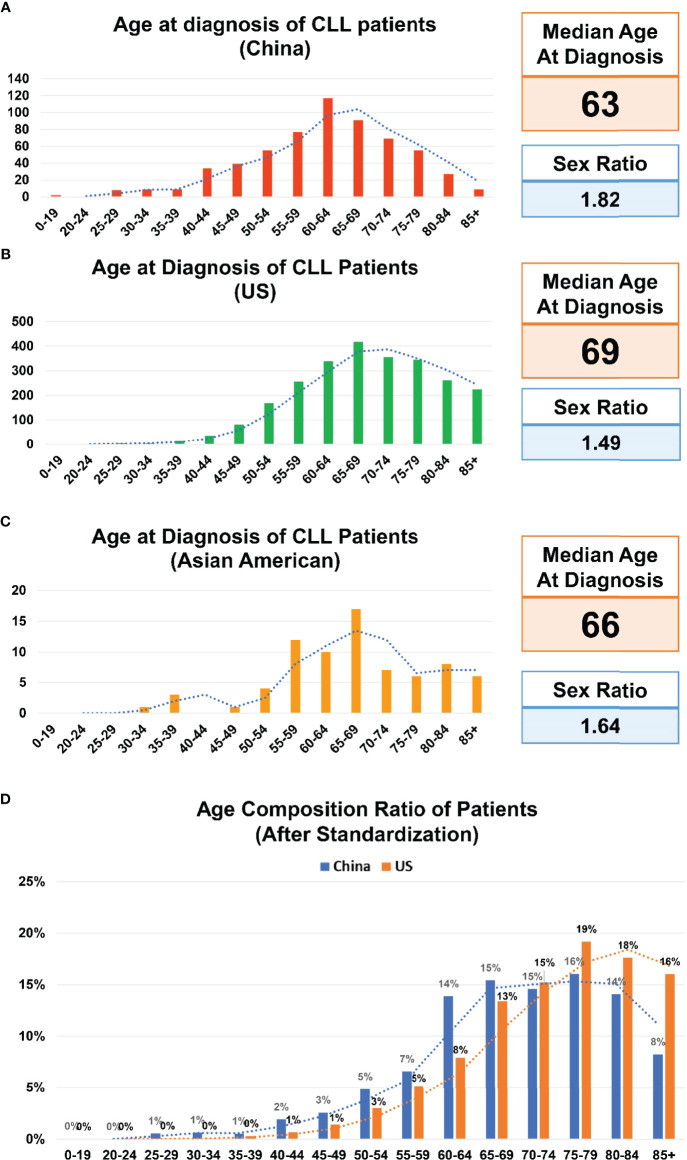
Age composition of CLL patients. **(A)** Age composition of Chinese patients with chronic lymphocytic leukemia (CLL) included in the Shandong Provincial Hospital CLL (SPHCLL) database from 2010 to 2021. **(B)** Age composition of CLL patients of predominately European descent registered in the Surveillance, Epidemiology, and End Results (SEER) database from 2010 to 2018. **(C)** Age composition of Asian Americans with CLL registered in the SEER database from 2010 to 2018. **(D)** Age composition ratio of CLL patients after standardization according to the age distributions of the United States (US) and China.

Between October 2010 and October 2021, the information on 601 patients with a diagnosis of CLL was included in the SPHCLL database. The median age at onset of CLL patients was 63 years old and the ratio of male/female was 1.82 (**Figure 1A**). 233 patients were divided into the young group with the mean age of 52 years old. 368 patients were divided into the elderly group with the mean age of 68 years old. Moreover, basic information on 2506 CLL patients of predominately European descent with the median age at diagnosis of 69 years old were obtained from the SEER database between 2010 and 2018 (**Figure 1B**). Clinical information of 74 Asian Americans with CLL were assessed from the SEER database between 2010 and 2018. Notably, the median age at onset of Asian Americans with CLL in the SEER database was 66 years old, which was the intermediate age between Chinese and persons of predominately European descent (**Figure 1C**). Researchers adjusted the age composition ratio of CLL patients according to population age distributions of the US and China. After adjustments, a trend toward younger patients was still observed in the SPHCLL cohort (**Figure 1D**).

**Figure 2:**
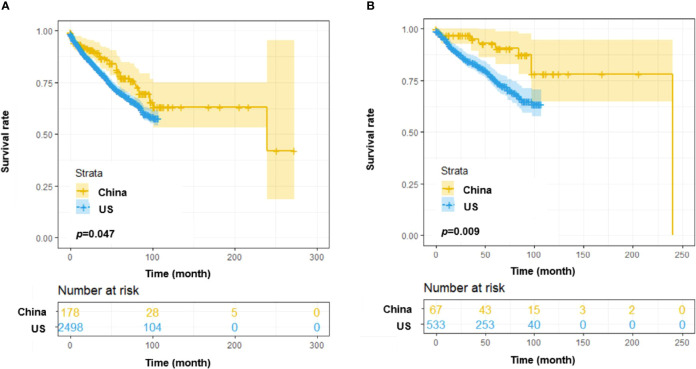
Survival analysis among CLL patients from the US and China. **(A)** Overall survival (OS) differences among patients from the SEER database and the Chinese SPHCLL database. **(B)** OS differences among young (<60 years old) patients from the SEER database and the Chinese SPHCLL database.

Moreover, the overall survival was compared between the SPHCLL cohort and the SEER cohort. Among CLL patients diagnosed between 2010 and 2018, The SPHCLL cohort presented prolonged overall survival than the SEER cohort (*p*=0.047; **Figure 2A**). Young patients in the SPHCLL cohort were featured by prolonged overall survival compared with young patients in the SEER cohort (*p*=0.009; **Figure 2B**). However, no significant difference was observed between the overall survival of elderly patients in the SPHCLL cohort and the SEER cohort (*p*=0.083). The baseline characteristics were compared between the SPHCLL cohort and SEER cohort. The age at diagnosis of the SPHCLL cohort was younger than that in the SEER cohort. 27.6% of the SPHCLL cohort have received chemotherapies. However, only 13.2% of the SEER cohort had records of receiving chemotherapies. Collectively, differences in biological features and treatment regimens might contribute to the survival difference between the SPHCLL cohort and the SEER cohort.

### Clinical-Pathological Characteristics

The general clinical data were compared between young and elderly SPHCLL patients. The proportions of CLL patients with combined diseases including coronary heart disease (*p*=0.010) and hypertension (*p*<0.001) were higher in the elderly group. But there were no statistically significant differences in the proportions of CLL patients with diabetes, tuberculosis or hepatitis B between the young and elderly groups. Del (13q) (37.4%) and trisomy 12 (15.0%) were the most frequent chromosomal aberrations detected by FISH. The frequencies of del (13q), trisomy 12, del (17p13) and del (11q22.3) showed no significant differences between young and elderly patients. IGHV unmutated status was observed in 34.3% of young patients and 32.6% of elderly CLL patients. And there was no significant difference in IGHV mutation status between young and elderly patients. Complex abnormal karyotype was observed in 12.0% of CLL patients. 13.5% of young CLL patients presented complex abnormal karyotype, and 11.3% of elderly CLL patients presented complex abnormal karyotype. No significant differences were observed between chromosome karyotype of young and elderly CLL patients.

Moreover, the association between laboratory parameters and age at onset was analyzed in this study. The levels of red blood cell (RBC) (*p*<0.001), hemoglobin (Hb) (*p*=0.023), hematocrit (*p*=0.011), platelet (*p*=0.019), platelet crit (*p*=0.034), albumin (*p*=0.047), alpha-1 antitrypsin (p=0.033), phosphorus (*p*=0.028), neutrophilic granulocyte percent (*p*=0.014) and estimated glomerular filtration rate (*p*<0.001) were significantly higher in young CLL patients, while the levels of mean corpuscular volume (*p*<0.001), mean corpuscular hemoglobin (*p*<0.001), red cell volume distribution width-SD (*p*<0.001), lymphocyte percentage (*p*=0.031), beta macroglobulin (*p*=0.031), creatinine (*p*=0.011), uric acid (*p*=0.004) and cystatin C (*p*=0.001) were decreased in young CLL patients (**Table 1**).

**Table 1:** Clinical-pathological parameters in young and elderly CLL patients.

Parameters	Young group (<60 years old)	Elderly group (≥60 years old)	*p*
Sex (male) (%)	150/233 (64.4)	238/368 (64.7)	0.941
Splenauxe (%)	62/112 (55.4)	96/174 (55.2)	0.976
Hepatomegaly (%)	9/107 (8.4)	12/171 (7.0)	0.816
Lymphadenectasis (%)	109/116 (94.0)	150/169 (88.8)	0.148
Smoking history (%)	47/111 (42.3)	60/172 (34.9)	0.212
Drinking history (%)	43/110 (39.1)	59/171 (34.5)	0.448
**Complications (%)**	129/155 (83.2)	231/252 (91.7)	0.011*
Coronary heart disease	6/89 (6.7)	61/169 (36.1)	<0.001***
Hypertension	28/95 (29.5)	94/167 (56.3)	<0.001***
Diabetes	18/95 (18.9)	45/162 (27.8)	0.134
Hepatitis B	9/87 (10.3)	8/147 (5.4)	0.195
Tuberculosis	4/84 (4.8)	6/146 (4.1)	0.815
**Binet stage (%)**			
A	22/107 (20.6)	43/175 (24.6)	0.213
B	47/107 (43.9)	58/175 (33.1)	
C	39/107 (36.4)	74/175 (42.3)	
**Rai stage (%)**			
0	8/107 (7.5)	13/172 (7.6)	0.912
I	27/107 (25.2)	40/172 (23.3)	
II	28/107 (26.1)	43/172 (25.0)	
III	16/107 (15.0)	22/172 (12.8)	
IV	28/107 (26.2)	54/172 (31.4)	
**FISH (%)**			
Normal	18/56 (32.1)	39/91 (42.9)	0.225
Del 17p13	7/56 (12.5)	13/91 (14.3)	0.810
Del 11q22.3	11/56 (19.6)	9/91 (9.9)	0.136
Del 13q	22/56 (39.3)	33/91 (36.3)	0.865
Trisomy 12	8/56 (14.3)	14/91 (15.4)	0.856
**IGHV mutation (unmutated) (%)**	12/35 (34.3)	15/46 (32.6)	0.874
**Chromosome karyotype**			
Complex abnormal karyotype	7/52 (13.5)	9/80 (11.3)	0.923
Uncomplex abnormal karyotype	8/52 (15.4)	12/80 (15.0)	
Normal karyotype	37/52 (71.2)	59/80 (73.8)	
**laboratory parameters (reference range)**			
RBC, (3.8-5.1) 10^12/L	4.17 ± 0.83	3.80 ± 0.95	<0.001***
Hb, (115-150) g/L	123.95 ± 27.27	116.82 ± 28.70	0.023*
HCT, (35-45) %	37.95 ± 7.08	35.80 ± 8.05	0.011*
MCV, (82-100) fL	91.65 ± 7.72	95.56 ± 8.97	<0.001***
RDW-SD, (39-46) fl	46.45 ± 6.13	50.33 ± 10.34	<0.001***
MCH, (27-34) pg	29.71 ± 3.00	30.88 ± 2.77	<0.001***
PLT, (125-300) 10^9/L	173.57 ± 98.93	149.81 ± 85.08	0.019*
PCT, %	0.18 ± 0.09	0.16 ± 0.09	0.034*
WBC, (3.5-9.5) 10^9/L	42.90 ± 96.84	49.33 ± 90.72	0.535
NEUT%, (40-75) %	28.55 ± 31.30	21.53 ± 18.42	0.027*
LYMPH%, (20-50) %	66.91 ± 25.25	72.78 ± 20.54	0.031*
AST, (13-35) U/L	26.02 ± 21.41	21.93 ± 11.90	0.054
ALT, (7-40) U/L	25.00 ± 30.04	16.58 ± 11.40	0.003**
ALB, (40-55) g/L	40.49 ± 5.94	39.42 ± 6.04	0.125
APO A, (1-2) g/L	1.03 ± 0.25	0.99 ± 0.23	0.163
LP (a), (0-0.3) g/L	0.26 ± 0.26	0.24 ± 0.22	0.361
AAT, (89-205) mg/dl	174.29 ± 61.31	153.47 ± 43.97	0.033*
CREA, (40-105) umol/L	68.77 ± 24.80	75.62 ± 21.62	0.011*
eGFR (>90)	102.83 ± 21.23	87.62 ± 15.25	<0.001***
URIC, (155-357) umol/L	301.69 ± 87.02	332.80 ± 93.94	0.004**
CysC, (0.54-1.15) mg/L	1.07 ± 0.42	1.31 ± 0.84	0.001**
PHOS, (0.83-1.48) mmol/L	1.22 ± 0.22	1.15 ± 0.21	0.005**
BMG, (1.0-3.0) mg/L	3.17 ± 2.04	3.69 ± 2.02	0.031*
LDH, (120-250) U/L	222.61 ± 108.45	239.66 ± 186.96	0.446

Bold p values represent statistical significance. *p < 0.05, **p < 0.01, ***p < 0.001.

AAT indicates alpha-1 antitrypsin; ALB, albumin; ALT, alanine aminotransferase; APO A, apolipoprotein A; AST, aspertate aminotransferase; BMG, beta macroglobulin; CREA, creatinine; CysC, cystation C; eGFR, estimated glomerular filtration rate; Hb, hemoglobin; HCT, hematocrit; LDH: lactate dehydrogenase; LP(a), lipoprotein(a); LYMPH%, lymphocyte percentage; MCH, mean corpuscular hemoglobin; MCV, mean corpuscular volume; NEUT%, neutrophilic granulocyte percent; PCT, platelet crit; PLT, platelet; RBC, red blood cell; RDW-SD, red cell volume distribution width-SD; WBC, white blood cell; PA, prealbumin; PHOS, phosphorus; and URIC, uric acid.

### Treatment and Response

The treatment options for CLL patients presented high diversity. Age, physical status, TP53 mutations and other factors can affect the choice of treatment options for CLL patients (1). Therefore, first-line treatment regimens were compared between young and elderly patients from the SPHCLL database. 32.19% of young CLL patients and 26.63% of elderly CLL patients have received treatment, and the first-line treatment regimens of CLL patients were listed in **Table 2**. In this study, the fludarabine and cyclophosphamide (FC) regimen (17.3%) was the most frequent treatment option among young CLL patients receiving first-line therapies, followed by the fludarabine, cyclophosphamide, and rituximab (FCR) regimen (14.7%). The most frequent first-line treatment option in elderly CLL patients receiving first-line therapies was FC regimen (26.5%), followed by chlorambucil (18.4%). There were no significant differences in the choice of first-line treatment regimens between the two groups.

**Table 2:** First-line treatment options and clinical efficacy evaluation in young and elderly CLL patients.

Parameters	Young group (<60 years old)	Elderly group (≥60 years old)	χ^2^	*p*
**First-line treatment options (%)**				
FC	13/75 (17.3)	26/98 (26.5)	2.756	0.103
FCR	11/75 (14.7)	9/98 (9.2)	1.249	0.338
CHOP	6/75 (8.0)	3/98 (3.1)	1.219	0.270
RCHOP	3/75 (4.0)	3/98 (3.1)	0.112	0.738
Ibrutinib	8/75 (10.7)	8/98 (8.2)	0.317	0.605
Bendamustine	5/75 (6.7)	2/98 (2.0)	1.302	0.254
Chlorambucil	10/75 (13.3)	18/98 (18.4)	0.794	0.411
**Clinical efficacy evaluation (%)**				
CR/PR	17/26 (65.4)	13/17 (76.5)		0.513
SD	4/26 (15.4)	1/17 (5.9)		0.633
PD	5/26 (19.2)	3/17 (17.6)		0.896
Refractory	5/26 (19.2)	5/17 (29.4)		0.481
Relapse	11/26 (42.3)	2/17 (11.8)		0.045*
**FC**				
CR/PR	5/7 (71.4)	10/12 (83.3)		0.603
PD	2/7 (28.6)	2/12 (16.7)		0.603

Bold p values represent statistical significance. *p < 0.05.

CHOP indicates cyclophosphamide, doxorubicin, vincristine and prednisone; CR, complete remission; FC, fludarabine and cyclophosphamide; FCR, ludarabine, cyclophosphamide and rituximab; PD, disease progression; PR, partial remission; RCHOP, rituximab plus cyclophosphamide, doxorubicin, vincristine and prednisone; SD, stable disease.

Efficacy assessments of first-line therapies were performed among young and elderly CLL patients respectively. Among young CLL patients who received first-line therapies, 65.4% of patients achieved complete remission/partial remission (CR/PR), 15.4% achieved stable disease (SD), 19.2% achieved disease progression (PD) and 42.3% relapsed. Among elderly patients who received first-line therapies, 76.5% achieved CR/PR, 15.4% achieved SD, 16.7% achieved PD and 11.8% relapsed. Compared with elderly CLL patients, the relapse rate was higher in young CLL patients (*p*=0.045). Moreover, FC regimen was the most frequent treatment option among CLL patients. However, there were no significant differences in the proportion of CR/PR, SD and PD between young and elderly patients receiving FC and FCR regimens (**Table 2**).

### Overall Survival and Progression-Free Survival

**Figure 3:**
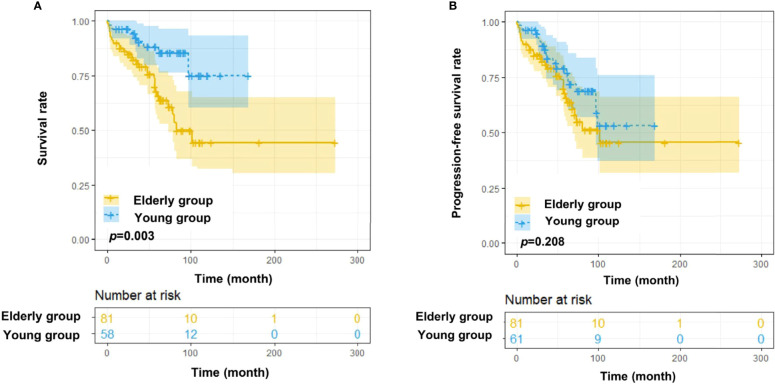
Survival analysis of Chinese CLL patients. **(A)** OS differences among young (<60 years old) and elderly (≥60 years old) Chinese CLL patients from the SPHCLL database. **(B)** Progression free survival (PFS) differences among young and elderly Chinese CLL patients from the SPHCLL database.

The elevated age at onset was significantly associated with decreased overall survival in the SPHCLL cohort (*p*=0.003; **Figure 3A**). However, there was no significant difference between the PFS of young and elderly patients in the SPHCLL cohort (**Figure 3B**). Furthermore, clinical-pathological characteristics with prognostic values in young and elderly patients were shown in **Table 3**. Multivariate Cox regression analysis showed independent prognostic values of prothrombin time (PT) (*p*=0.104) and RBC (*p*=0.039) in young CLL patients. Hb (*p*<0.001), globulin (GLO) (*p*<0.001) and blood urea nitrogen/creatinine (BUN/CREA) (*p*=0.001) were identified as independent prognostic factors in elderly CLL patients.

**Table 3:** Univariable and multivariable analyses of OS in young and elderly CLL patients.

Characteristics	Young group (<60 years old)					Elderly group (≥60 years old)				
	Univariate analysis		Multivariate analysis			Univariate analysis		Multivariate analysis		
	HR (95% CI)	*p*	Coef	HR (95% CI)	*p*	HR (95% CI)	*p*	Coef	HR (95% CI)	*p*
**Hb**	0.971 (0.951-0.991)	0.004**	NS	NS	NS	0.973 (0.961-0.985)	<0.001***	-0.030	0.970 (0.955-0.985)	<0.001***
**RBC**	0.327 (0170-0.629)	0.001**	-0.839	0.432 (0.195-0.960)	0.039*	0.460 (0.316-0.670)	<0.001***	NS	NS	NS
**PT**	1.326 (1.047-1.679)	0.019*	0.155	1.168 (0.968-1.408)	**0.104**	1.115 (0.942-1.319)	0.205	ND	ND	ND
**ALB**	0.915 (0.850-0.985)	0.019*	NS	NS	NS	0.907 (0.853-0.984)	0.002**	NS	NS	NS
**GLO**	1.038 (0.938-1.147)	0.470	ND	ND	ND	1.038 (1.001-1.077)	0.045*	-0.095	1.099 (1.044-1.158)	<0.001***
**HDL-C**	0.032 (0.003-0.307)	0.003**	NS	NS	NS	0.198 (0.041-0.966)	0.045*	NS	NS	NS
**APO A**	0.048 (0.007-0.311)	0.048*	NS	NS	NS	0.086 (0.019-0.388)	0.001**	NS	NS	NS
**BUN**	1.060 (1.001-1.123)	0.046*	NS	NS	NS	1.146 (1.026-1.279)	0.015*	NS	NS	NS
**BUN/CREA**	1.011 (0.999-1.024)	0.072	ND	ND	ND	1.019 (1.007-1.030)	0.001**	-0.028	1.028 (1.011-1.045)	0.001**
**RBP**	0.986 (0.936-1.039)	0.592	ND	ND	ND	0.965 (0.935-0.996)	0.028*	NS	NS	NS

Bold p values represent statistical significance. *p < 0.05, **p < 0.01, ***p < 0.001.

BUN indicates blood urea nitrogen; BUN/CREA, blood urea nitrogen/creatinine; 95% CI indicates 95% confidence interval; GLO, globulin; HDL-C, high-density lipoproteincholesterol; ND, not done; NS, not signficant; PT, prothrombin time; RBP, retinol binding protein; ALB, APO A, Hb and RBC are explained in **Table 1**.

### *PR* and *HBG* Risk Scoring Systems for CLL Patients in Different Age Groups

**Figure 4:**
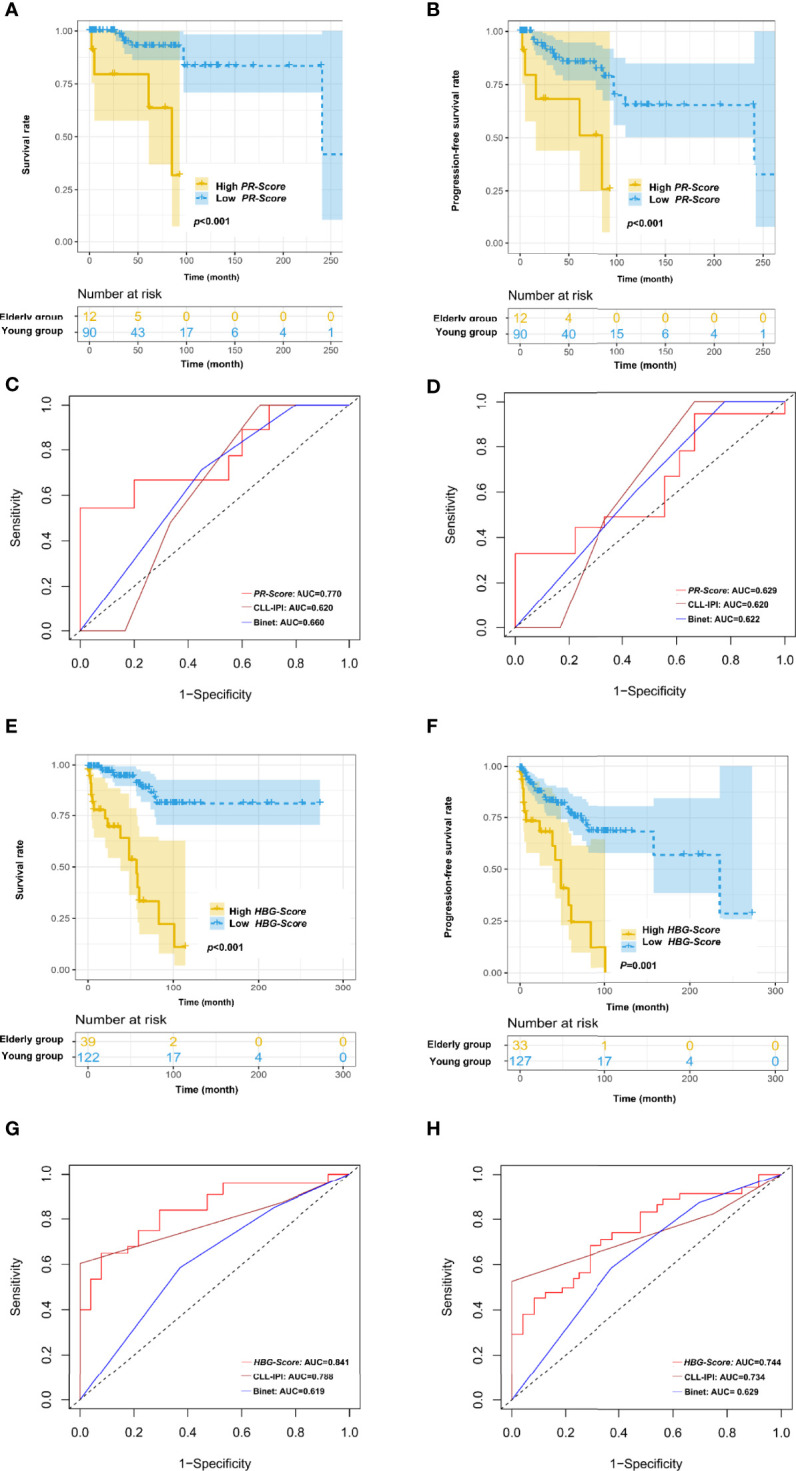
Novel risk score models for young (<60 years old) and elderly (≥60 years old) CLL patients. **(A)** Increased *PR-Score* significantly correlated with shortened OS in young patients. **(B)** Increased *PR-Score* significantly correlated with shortened PFS in young patients. **(C)** *PR-Score* model showed advanced prognostic ability than international prognostic index for CLL (CLL-IPI) and Binet stage in predicting OS of young patients. **(D)** *PR-Score* model showed advanced prognostic ability than CLL-IPI and Binet stage in predicting PFS of young patients. **(E)** Increased *HBG-Score* significantly correlated with shortened OS in elderly patients. **(F)** Increased *HBG-Score* significantly correlated with shortened PFS in elderly patients. **(G)** *HBG-Score* model showed advanced prognostic ability than CLL-IPI and Binet stage in predicting OS of elderly patients. **(H)** *HBG-Score* model showed advanced prognostic ability than CLL-IPI and Binet stage in predicting PFS of elderly patients.

To optimize risk assessment of CLL patients in different age groups, new risk score systems were developed for young and elderly patients respectively. Based on the results of multivariate Cox regression analysis, PT and RBC were selected for establishing the *PR-Score* model in young patients: *PR-Score* = (0.155) * PT + (-0.839) * RBC. The cut-off point of *PR-Score* was determined as -0.47299. The Kaplan-Meier curve showed that increased *PR-Score* was significantly associated with shortened OS in young patients (*p*<0.001; **Figure 4A**). Further investigations revealed a statistically significant association between *PR-Score* and PFS in young patients (*p*<0.001; **Figure 4B**). The *PR-Score* model has shown advanced prognostic capacity to predict OS and PFS of CLL patients than international prognostic index for CLL (CLL-IPI) and Binet stage system in young patients (**Figures 4C, D**).

Hb, GLO and BUN/CREA were selected for establishing the *HBG-Score* model in elderly patients: *HBG-Score* = (-0.03) * Hb + (0.028) * BUN/CREA + (0.095) * GLO. The cut-off point of *HBG-Score* was determined as 2.14022. The increased *HBG-Score* was significantly associated with shortened OS (*p*<0.001) and PFS (*p*=0.001) in elderly patients (**Figures 4E, F**). The *HBG-Score* model has also shown better prognostic ability to predict OS and PFS of CLL patients than CLL-IPI and Binet stage system in elderly patients (**Figures 4G, H**).

## Discussion

CLL patients from China are characterized by an earlier age at onset compared with Western countries. To explore the association between the distinct age distribution and clinical characteristics of Chinese CLL patients, this study illustrated the age-related differences in clinical-pathological features and outcomes. The *PR-Score* model for young patients and *HBG-Score* model for elderly patients were developed to optimize the risk stratification system of CLL patients in different age groups.

The epidemiologic features of CLL are significantly different between Asians and persons of predominately European descent (19). The median age at onset of patients from the SPHCLL database was 63 years old, which was similar to results in other centers of China (20). However, it was earlier than the median age at onset of CLL in Western countries, which reflected epidemiologic distinctions between different regions and races (21). Although different population age distributions in China and the US may have impacts on the median age at onset (22), after adjustments according to population age distributions in China and the US, the trend toward earlier age at onset can still be observed in the SPHCLL cohort. Intriguingly, Asian Americans are characterized by an intermediate age at onset of CLL between Chinese and persons of predominately European descent. The different age distributions of CLL patients between China and Western countries are not completely determined by genetic factors. Environmental factors and epigenetic modifications may also have impacts on the age at diagnosis of CLL.

The general clinical characteristics were compared between the SPHCLL cohort and patients of predominately European descent. The frequencies of chromosomal aberrations detected by FISH in the SPHCLL cohort were similar to that in western countries. In the SPHCLL cohort, the frequency of chromosomal aberrations detected by FISH was 61.2%: del (13q) was 39%, trisomy 12 was 15%, del (17p13) was 14%, and del (11q22.3) was 14%. Among western populations, the frequency of del (13q) was 26-46%, trisomy 12 was 13-21%, del (17p13) was 8-12%, and del (11q22.3) was 12-15% (23–25). Moreover, 27.6% of the SPHCLL cohort have received chemotherapies, and 13.2% of the SEER cohort have received chemotherapies. Intriguingly, the SPHCLL cohort presented prolonged overall survival compared with the SEER cohort. The younger age at diagnosis and increased rate of receiving chemotherapies of the SPHCLL cohort could contribute to the survival difference between the SPHCLL cohort and the SEER database. Although the cytogenetic abnormalities and specific treatment regimens were not available to access in the SEER database, these factors might also have impacts on the results of survival analyses.

Moreover, elderly CLL patients in China received FC regimen and FCR regimen more frequently than CLL patients in western countries. The guidelines for diagnosis and treatment of chronic lymphocytic leukemia/small lymphocytic lymphoma in China (2015 edition) recommended bendamustine for the treatment of elderly CLL patients firstly. Although the U.S. Food and Drug Administration (FDA) approved bendamustine for the treatment of CLL in 2010, bendamustine was not approved by China Food and Drug Administration (CFDA) for the treatment of CLL until 2018. Therefore, FC regimen and FCR regimen used to be the most frequent regimens among elderly CLL patients.

CLL patients in China have shown distinct age distribution compared with Western countries. Therefore, it is necessary to explore the age-related clinical-pathological features and outcomes of Chinese CLL patients, which may provide insights into the more advanced treatment for CLL (26, 27). Although the OS was prolonged in young patients, there was no significant difference in the PFS of young and elderly patients. The phenomenon can be attributed to the more progressive disease status of young CLL patients. Moreover, the rate of relapse was superior in young CLL patients than elderly patients.

Univariate Cox regression analysis demonstrated that apo A, albumin and high-density lipoprotein cholesterol (HDL-C) were prognostic factors in age subgroups. The above contents indicate the importance of abnormal lipid metabolism and albumin metabolism in CLL patients. Studies on the pathogenesis of CLL are increasingly focusing on aberrant lipid metabolism (28). The aberrant regulation of apo A could be attributed to CLL pathogenesis. Previous studies have provided clues to the mechanisms underlying the prognostic values of apo A and HDL-C (29, 30). Proteolytic products of apo A participate in anti-angiogenic and anti-tumoral activities (31). Cholesterol and other cellular lipids released from cells are accepted by apo A-I, promoting the formation of nascent high density lipoprotein (32). The regulation of metabolism and homeostasis of cholesterol are lost in CLL cells.

Serum albumin is a protein produced by the liver, which plays a role in maintaining plasma colloid osmotic pressure, participating in redox reactions *in vivo*, maintaining capillary permeability, and controlling cell apoptosis (33, 34). It is also an indicator of nutritional and inflammatory status (35, 36). Reduced serum concentration of albumin was associated with poor prognosis in multiple cancers including non-small cell lung cancer, prostate cancer and gastric cancer (37, 38). Studies have shown that chronic inflammation is a favorable feature of cancer, which is of great significance for the progression of CLL (39, 40). The increase of microvessel permeability during chronic inflammation causes abnormal distribution of albumin and accelerates albumin degradation (41). Previous studies demonstrated that inflammatory markers including high C-reactive protein-to-albumin ratio and low albumin-to-fibrinogen ratio predicted poor prognostic of CLL (42, 43).

In conclusion, CLL patients from China are relatively younger than CLL patients of predominately European descent. The present study analyzed the clinical-pathological characteristics and outcomes of young and elderly CLL patients from China. Young patients presented a more progressive disease course than elderly patients. A novel prognostic model named *PR-Score* was developed for accurate prediction of outcomes in young CLL patients, and an *HBG-Score* was developed for prediction of outcomes in elderly patients. This investigation provided a comprehensive profile of young and elderly Chinese CLL patients, emphasizing the accurate risk assessment of CLL patients in various age subgroups. The neonatal score systems are expected to optimize the treatment regimens for CLL patients in different age groups.

## Data Availability Statement

The original contributions presented in the study are included in the article. Further inquiries can be directed to the corresponding authors.

## Author Contributions

XW and YZ designed the study protocol, analyzed the data and edited the manuscript; ZT and ML analyzed the data and wrote the first draft of the manuscript; XF, XZ, PL, YL, LZ and FL made contributions to acquisition and interpretation of data; and all authors reviewed the manuscript, approved the final version, decided to publish this report and vouch for the data accuracy and completeness.

## Funding

This study was funded by National Natural Science Foundation (No. 82000195, No. 82070203 and No. 81770210); Key Research and Development Program of Shandong Province (No. 2018CXGC1213); Translational Research Grant of NCRCH (No. 2021WWB02, No. 2020ZKMB01); Taishan Scholars Program of Shandong Province; Shandong Provincial Natural Science Foundation (No. ZR2020QH094); Shandong Provincial Engineering Research Center of Lymphoma; Technology Development Project of Jinan City (No. 202019182); Academic Promotion Programme of Shandong First Medical University (No. 2019QL018, No. 2020RC007); Shandong Provincial Hospital Youth Talent Plan.

## Conflict of Interest

The authors declare that the research was conducted in the absence of any commercial or financial relationships that could be construed as a potential conflict of interest.

## Publisher’s Note

All claims expressed in this article are solely those of the authors and do not necessarily represent those of their affiliated organizations, or those of the publisher, the editors and the reviewers. Any product that may be evaluated in this article, or claim that may be made by its manufacturer, is not guaranteed or endorsed by the publisher.
